# Comparison of Visual Outcomes Between Manual Phacoemulsification and Femtosecond Laser-Assisted Cataract Surgery

**DOI:** 10.7759/cureus.103921

**Published:** 2026-02-19

**Authors:** Megan Sharp, Gabriela Carrillo, Adam Salama, Rahul Garg, Eric B Johnson

**Affiliations:** 1 Medicine, Alabama College of Osteopathic Medicine, Dothan, USA; 2 Medicine, University of Miami Miller School of Medicine, Miami, USA; 3 Research, Alabama College of Osteopathic Medicine, Dothan, USA; 4 Anatomy and Molecular Medicine, Alabama College of Osteopathic Medicine, Dothan, USA

**Keywords:** cataract lens, femtosecond laser-assisted cataract surgery (flacs), manual small incision cataract surgery, phacoemulsification cataract surgery, uncomplicated cataract surgery

## Abstract

Objective

Our study compared recovery outcomes between manual and femtosecond laser-assisted cataract surgery by measuring visual acuity, intraocular pressure, and inflammation through pachymetry testing. We hypothesized that laser-assisted procedures would have improved postoperative results when compared to manual cataract surgery.

Design

We performed a prospective cohort study. Patients undergoing either manual or femtosecond laser-assisted cataract surgery by a single surgeon were followed through preoperative evaluation and three postoperative visits at one day, one week, and one month, documenting values of visual acuity, intraocular pressure, and pachymetry results.

Subjects, participants, and controls

We followed patients who fit the criteria for cataract removal, as diagnosed by a board-certified ophthalmologist, with eye surgeries divided into two groups: femtosecond laser-assisted surgery (n=35) and manual surgery (n=63).

Methods

All patients underwent either manual or femtosecond laser-assisted cataract surgery by the same surgeon. Each eye was evaluated preoperatively and at one day, one week, and one month postoperatively.

Main outcome measures

The outcomes measured were postoperative visual acuity, intraocular pressure, and pachymetry (as a proxy for inflammation) in order to assess recovery outcomes. Repeated measures ANOVA was used to analyze the mean changes in visual recovery outcomes.

Results

No statistically significant difference was found between the femtosecond laser-assisted surgery versus manually performed cataract surgery. However, the average for the two visual outcomes increased despite the statistics not being significant. Future studies are warranted with a larger sample size and more control variables.

Conclusions

This study sought to determine the variances in recovery outcomes between manual cataract surgery and femtosecond laser-assisted surgery by assessing visual acuity, corneal edema, and intraocular pressure. Our findings suggest that there was no significant statistical difference in outcomes when comparing patients who elected to undergo manual cataract surgery versus femtosecond laser-assisted surgery.

## Introduction

Since its introduction in 2008, the femtosecond laser has seen increasing use in performing cataract removal procedures [1]. Cataracts are the leading cause of visual impairment globally, and the USA had a recorded incidence of 19.6 million cases in 2021 [2]. Internationally, over 20 million cataract surgeries are performed each year, and the rate is expected to increase due to the aging population [1,3]. In its use in cataract surgery, the femtosecond laser has the advantage of creating a more centered and consistent capsulorhexis, aiding the surgeon to soften and remove the nucleus [1]. The laser then sends a sharp, precise beam that creates plasma surrounding the lens and allows for a shock wave to develop [4]. This allows for the surgeon to disrupt the lens without harming surrounding tissue [4]. As laser surgery becomes more common, an increasing number of younger patients are requesting cataract surgery because of the visual acuity benefits and higher precision [1,5]. The laser makes a more predictable, well-shaped corneal incision and more precisely treats corneal astigmatism [4,6]. Manual phacoemulsification requires the surgeon to create corneal incisions with a handheld blade, possibly leading to imprecise length and structure [4].

Our research focused on comparing the outcomes of visual acuity, pachymetry, and intraocular pressure (IOP) between manual and femtosecond laser-assisted cataract surgeries. Previous research comparing the two surgical methods involves data from multiple surgeons, which could bias the results [7]. Our research focused on comparing both surgical methods performed by a single physician in order to remove any surgeon-related bias. Our study was prospective and followed patients through their preoperative appointment and three postoperative appointments at one day, one week, and one month. The primary goal of this study was to compare visual recovery outcomes between manual and laser-assisted cataract surgeries. Currently, research comparing health effects between manual versus laser surgery has not found statistically significant results [8]. Our hypothesis posits that laser-assisted cataract surgery will lead to a reduction in recovery time for participants, through evaluation of vision improvement, IOP, and inflammation, as measured through pachymetry.

## Materials and methods

Methods

A prospective cohort study was conducted at the office of S. Daniel Salama MD PA from March 5, 2024, through March 4, 2025. Participants in the study consisted of patients who met the criteria for cataract removal as diagnosed by a board-certified ophthalmologist. These patients independently selected either the manual or femtosecond laser-assisted method for their cataract procedure. All patients who consented for the study were included in the study with no exclusion variables. Certain patients' follow-up data were excluded from the study due to a clerical oversight that resulted in missing charts from some appointments.

All procedures and evaluations were performed by a single board-certified ophthalmologist throughout the duration of the study. Each patient attended four appointments: one preoperative evaluation and three postoperative follow-up visits conducted at one day, one week, and one month after surgery. During these visits, visual improvement, corneal edema, and IOP were assessed to evaluate surgical outcomes. Visual acuity was measured using the Snellen eye chart at a distance of 20 feet. Corneal pachymetry assessed edema based on average temporal corneal thickness. IOP was measured via applanation tonometry using Proparacaine Hydrochloride Ophthalmic Solution USP 0.5% eye drops for numbing.

Data analysis

We calculated mean changes in the post-surgical outcomes of visual acuity, pachymetry, and IOP. Repeated measures using analysis of variance (ANOVA) was used to analyze the changes in visual acuity, pachymetry, and IOP between per-operative baseline and the three postoperative follow-up visits at one day, one week, and one month for both manual and femtosecond laser at a p-value of 0.05.

## Results

**Figure 1:**
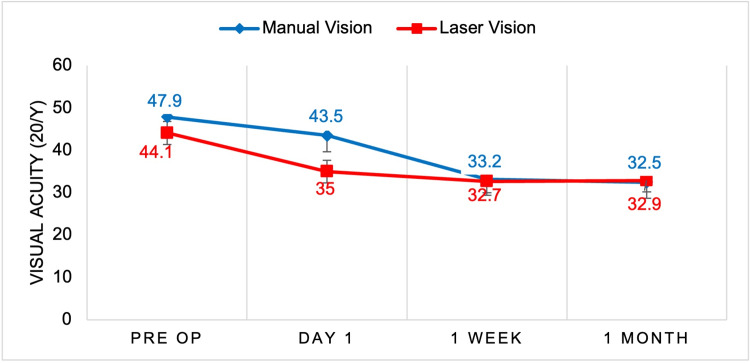
Manual versus laser cataract surgery mean comparative analysis of vision Visual acuity (Y-axis) of each patient was measured before surgery, and one day, one week, and one month after surgery (X-axis). Results from manual cataract surgery (blue) were compared to femtosecond laser-assisted cataract surgery (red). All time points showed no significant difference (p>0.05).

**Figure 2:**
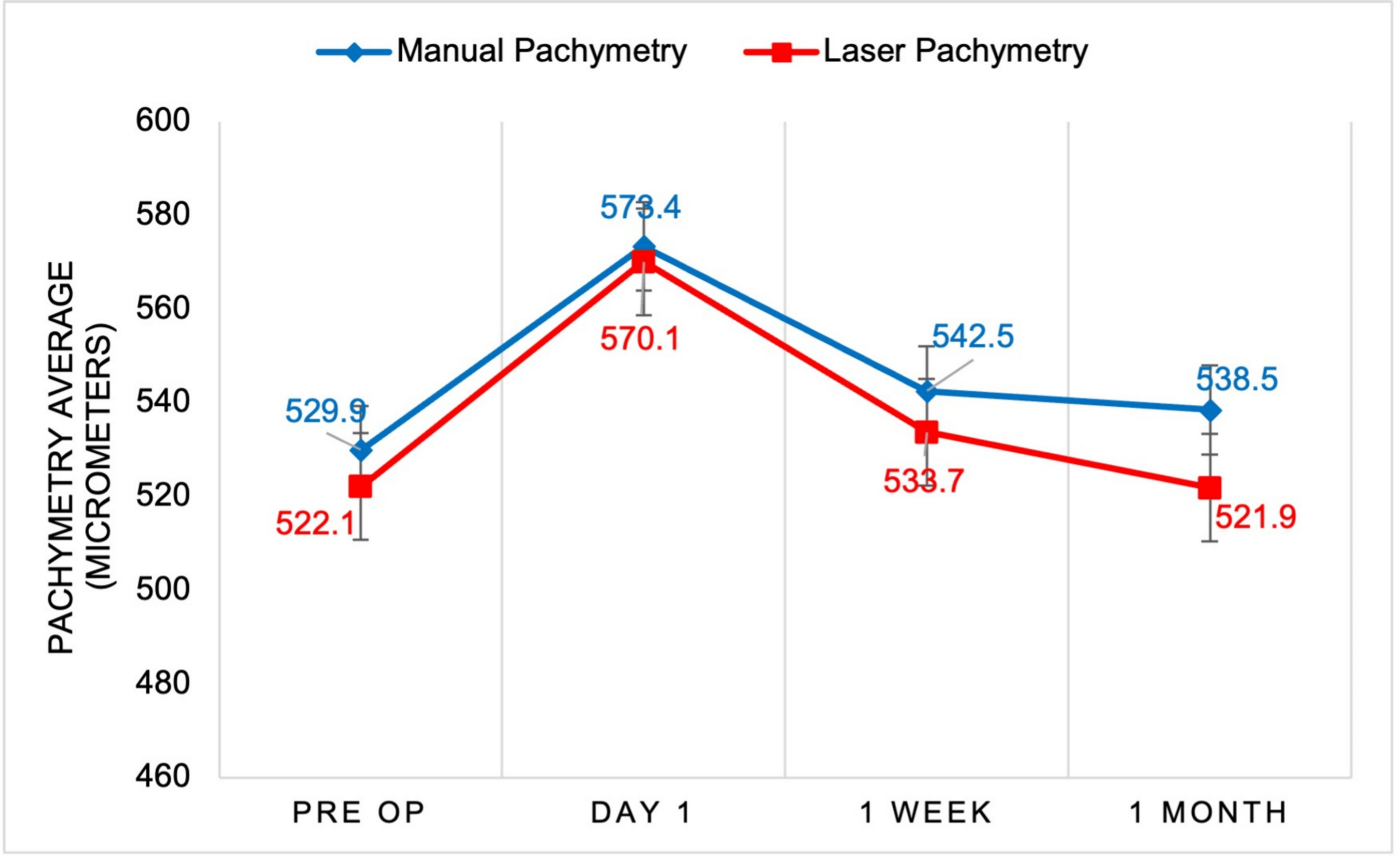
Manual versus laser cataract surgery mean comparative analysis of pachymetry Pachymetry (Y-axis) of each patient was measured before surgery, and one day, one week, and one month after surgery (X-axis). Results from manual cataract surgery (blue) were compared to femtosecond laser-assisted cataract surgery (red). All time points showed no significant difference (p>0.05).

**Figure 3:**
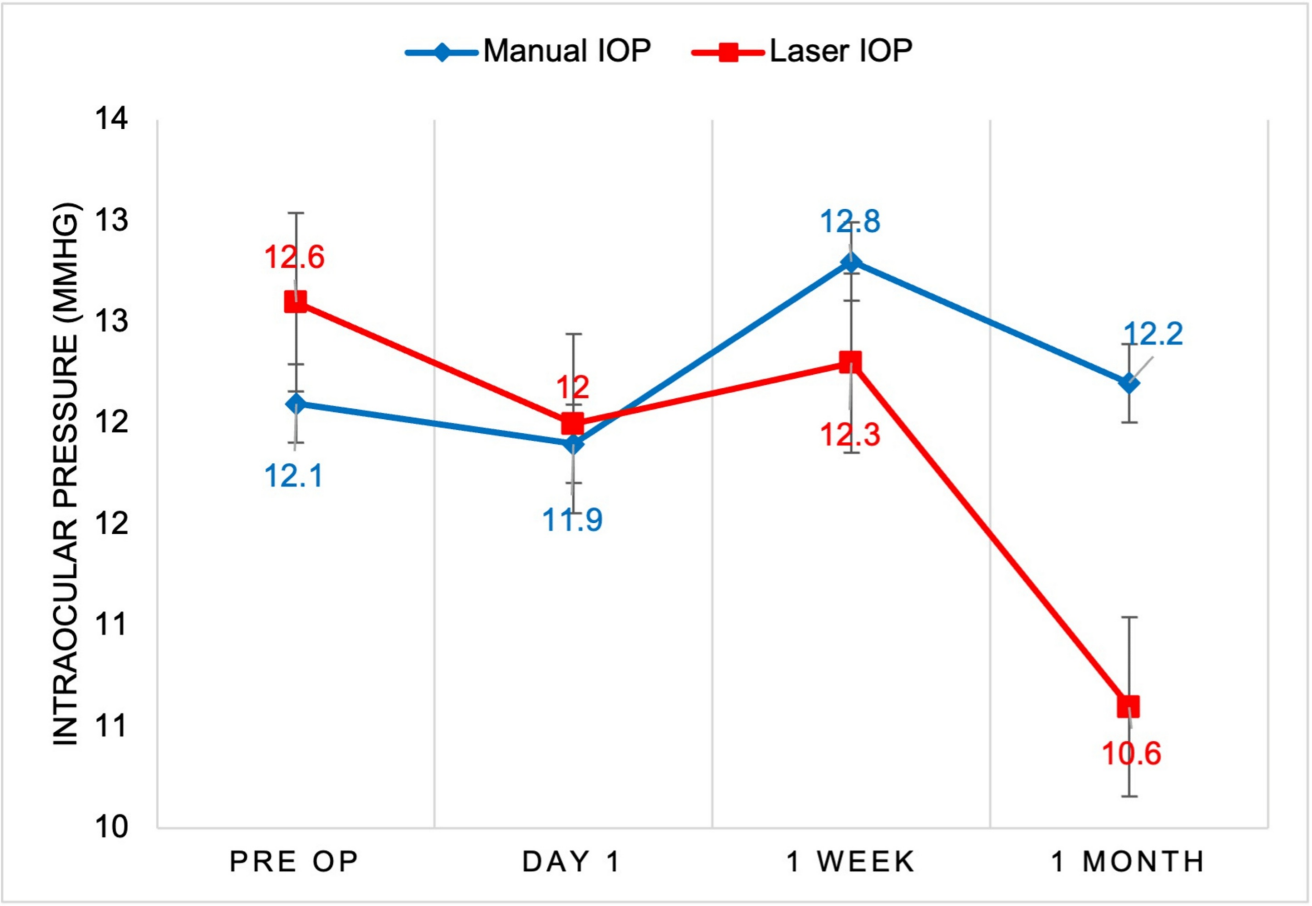
Manual versus laser cataract surgery mean comparative analysis of IOP IOP (Y-axis) of each patient was measured before surgery, and one day, one week, and one month after surgery (X-axis). Results from manual cataract surgery (blue) were compared to femtosecond laser-assisted cataract surgery (red). All time points showed no significant difference (p>0.05). IOP, intraocular pressure

A total of 98 patients were included in the study, with 62.29% (n=63) undergoing manual cataract surgery and 35.71% (n=35) undergoing femtosecond laser-assisted surgery. The sample included 62.24% (n=61) females and 37.76% (n=37) males, with surgeries performed on 49.48% (n=48) left eyes and 50.52% (n=49) right eyes. The blue line in each figure represents manually operated cataract surgery, whereas the red line represents the femtosecond laser technique. The figures show the progression of visual acuity, pachymetry, and IOP through the preoperative and three postoperative evaluations. In Figure 1, we saw an overall increase in visual acuity using the manual and laser techniques. Both techniques saw an overall improvement in visual acuity at the conclusion of the postoperative appointment near the value of 20/30 on the Snellen chart. In Figure 2, we compared the mean pachymetry values, representing inflammation of the eye. For both techniques, we saw an initial increase immediately after surgery on postoperative day 1, ranging from 570 and 573 micrometers. With both techniques, the inflammation gradually declines back to each patient’s baseline at the one-month appointment. In Figure 3, we saw an initial decline in IOP for both manual and laser at day 1. We then saw a slight rise in IOP by one week and another decline at one month. There were no statistically significant differences comparing these three variables between the manual versus laser techniques.

Although we observed a trend for increased visual acuity in the femtosecond laser-treated group one day post-surgery, there were no statistical differences between the laser and manual treatment groups (Figure 1). Furthermore, we did not find a statistical difference in pachymetry results between the laser and manual treatment groups at any of the time points measured (Figure 2). The last parameter measured was IOP. Although there was a trend for decreased IOP in the laser group compared to the manual group, there were no statistical differences between the treatment groups at any of the time points measured (Figure 3). Despite the statistics not being significant, the average for the two visual outcomes increased. Future studies with a larger sample size and more control variables are warranted.

## Discussion

This study sought to compare recovery outcomes between manual cataract surgery and femtosecond laser-assisted surgery by assessing visual acuity, corneal edema (as measured with pachymetry), and IOP. Our findings suggest that the average visual outcomes for pachymetry and IOP were improved with femtosecond laser-assisted surgery without statistical significance as compared to manual cataract surgery.

This study could assist patients to make an informed decision if they want femtosecond laser-assisted surgery or manually performed cataract surgery. Our data shows there was no statistical difference between treatments, but in our specific patient population, we did not observe any harm associated with performing laser surgery over manual. Acknowledging that the Food and Drug Administration approved the use of the femtosecond laser in 2010, this procedure is relatively new and patients may have concerns about safety and efficacy [9]. This study may alleviate concerns of safety that a patient may have when choosing between cataract removal techniques. While our analysis was limited to evaluating outcomes between manual and femtosecond laser-assisted cataract surgery, it is important to consider different outcomes based on comorbidities, age, and sex. Although there are no current studies that take these variables into consideration when comparing the outcomes between manual and femtosecond laser-assisted cataract surgery, comorbidities such as diabetes have been noted to affect the outcome of cataract surgery in general [10]. A recent systematic review found that diabetic patients with cataracts pose a significant challenge during both surgery and postoperative recovery [10]. A separate study concluded that patients with diabetes are reported to have more preoperative, intraoperative, and postoperative complications [11].

Currently, the European Journal of Ophthalmology does not have a general recommendation of one method of cataract surgery over another, as no large-scale study has shown either to be superior in outcomes [12]. This is supported by recent analyses of the literature, which have shown no advantage to using either femtosecond laser-assisted or manual cataract surgery, including a 2017 narrative review [13]. This is one of the few investigations that rely primarily on a single board-certified ophthalmologist, which allowed us to assess the recovery outcomes without variations in surgeon performance. Further investigation is needed to compare other variables such as total surgical time, precision of the capsulotomy, and late complications such as incidence of retinal detachment. These variables, among others, could shed light on whether using femtosecond laser has improved efficacy and recovery outcomes when compared to manually performed cataract surgery. Given that this study uses the outcomes of a single surgeon and surgery center, with a lower number of patients, further research into the efficacy is needed in order to improve the body of evidence and help direct patient decision-making.

## Conclusions

In this prospective comparison of manual versus femtosecond laser-assisted cataract surgery performed by a single surgeon, we found no statistically significant differences in postoperative visual acuity, pachymetry, or IOP between the two surgical techniques. Although femtosecond laser-assisted surgery demonstrated trends toward improved pachymetry and IOP outcomes, these differences did not reach statistical significance, suggesting comparable short-term recovery profiles between approaches in our patient population.

Importantly, no increased risk or adverse outcomes were observed with femtosecond laser-assisted cataract surgery, supporting its safety when compared to conventional manual phacoemulsification. Given the relative novelty of femtosecond laser technology and ongoing concerns regarding its clinical value and cost-effectiveness, these findings provide reassurance that laser-assisted cataract surgery does not compromise patient outcomes when compared to traditional techniques. Further large-scale studies incorporating diverse patient populations and additional outcome measures including surgical efficiency, capsulotomy precision, long-term visual stability, and postoperative complications are warranted to better define the clinical role of femtosecond laser technology and identify patient populations that may derive the greatest benefit.
